# Genetic Changes in Mastocytes and Their Significance in Mast Cell Tumor Prognosis and Treatment

**DOI:** 10.3390/genes15010137

**Published:** 2024-01-22

**Authors:** Szymon Zmorzynski, Aleksandra Kimicka-Szajwaj, Angelika Szajwaj, Joanna Czerwik-Marcinkowska, Jacek Wojcierowski

**Affiliations:** 1 Laboratory of Genetics, Academy of Zamosc, 22-400 Zamosc, Poland; 2 DVM Veterinary Hospital AMVET, 31-328 Krakow, Poland; aleksandra.kimicka@gmail.com; 3 St. Luke’s Hospital in Tarnow, 33-100 Tarnow, Poland; szajwaja@gmail.com; 4 Institute of Biology, Jan Kochanowski University, 25-369 Kielce, Poland; marcinko@kielce.com.pl; 5 Laboratory for Genetic Testing, 20-143 Lublin, Poland; alinawojcierowska@gmail.com

**Keywords:** mastocytes, mast cells, mastocytosis, mastocytoma, mutations, genes, microRNA

## Abstract

Mast cell tumors are a large group of diseases occurring in dogs, cats, mice, as well as in humans. Systemic mastocytosis (SM) is a disease involving the accumulation of mast cells in organs. *KIT* gene mutations are very often seen in abnormal mast cells. In SM, high KIT/CD117 expression is observed; however, there are usually no *KIT* gene mutations present. Mastocytoma (MCT)—a form of cutaneous neoplasm—is common in animals but quite rare in humans. KIT/CD117 receptor mutations were studied as the typical changes for human mastocytosis. In 80% of human cases, the *KIT* gene substitution p.D816H was present. In about 25% of MCTs, metastasis was observed. Changes in the gene expression of certain genes, such as overexpression of the *DNAJ3A3* gene, promote metastasis. In contrast, the *SNORD93* gene blocks the expression of metastasis genes. The panel of *miR-21-5p*, *miR-379*, and *miR-885* has a good efficiency in discriminating healthy and MCT-affected dogs, as well as MCT-affected dogs with and without nodal metastasis. Further studies on the pathobiology of mast cells can lead to clinical improvements, such as better MCT diagnosis and treatment. Our paper reviews studies on the topic of mast cells, which have been carried out over the past few years.

## 1. Introduction

Mast cell tumors are a large group of diseases occurring in dogs, cats, mice, as well as in humans. They can be relatively benign or malignant tumors. The prognosis in benign cases can be somewhat approximated, while in malignant cases, the prognosis is rather poor. The form of cutaneous mast cell tumor—mastocytoma—is particularly common in dogs. It accounts for 16–21% of all canine skin tumors and can cause considerable frustration and sadness for afflicted dogs [1,2].

## 2. Characteristics of Mastocytes

Mast cells (MCs) were first identified by Paul Ehrlich in 1878 [3]. MCs are spherical cells in the cytoplasm, which contain a significant number of secretory granules. Cells similar to mast cells are basophils, which are slightly smaller in size than MCs (8 μm); their cytoplasm has fewer granules; their granules do not contain tryptase; their cell membrane has no KIT receptor but contains the FcεR1 receptor. Basophil precursors mature in the bone marrow under the influence of Il-3 [4]. The granules stain metachromatically with toluidine blue, safranin, or berberine sulfate. The function of MCs, according to Ehrlich, was initially linked to the tissue nutrition system (hence the name “mast cells”). After detecting the release of heparin and histamine from MCs in anaphylactic shock [5], mast cells have been found to play a role in type I hypersensitivity reactions (allergy) [6]. MCs play a role in regulating allergic and inflammatory processes, secrete mediators for both innate and acquired immunity [7], play a role in the maintenance of immune homeostasis [8], and play a role in wound healing processes [9,10]. MCs, together with dendritic cells (DCs) and monocytes, are the first cells of the immune system, which interact with foreign antigens. After appropriate stimulation, MCs can re-enter the cell cycle and proliferate. The increased recruitment and local maturation of mast cell progenitors can also contribute to increasing MC populations in tissues [11]. During individual biological responses, MCs can function as effector cells, immunoregulatory cells, or both [12]. MC effector functions are associated with (I) removal of pathogens via phagocytosis and/or secretion of antimicrobial proteins; (II) degradation of toxic endogenous peptides and venom components; (III) increased vascular permeability (e.g., via histamine secretion); (IV) stimulation of bronchial smooth muscle cell contraction (e.g., via leukotriene C4); (V) stimulation of collagen synthesis by fibroblasts (e.g., via tryptase) [12].

Mast cell granules contain factors and substances such as polyamines, amines (serotonin, dopamine, and histamine), enzymes (cathepsins, β-hexosaminidase, arylsulfatase, heparanase, caspase, angiogenin, and kallikreins), proteases (metalloproteinases, chymase, granzyme B, carboxypeptidase A, tryptase), proteoglycans (heparin, serglycine, and chondroitin sulfate), cytokines (TNF-α, TNF-γ, β-FGF, Il-4, SCF, and most interleukins), chemokines (CCL2, CCL5, CCL7, CCL11, and MCP4), peptides (endorphin, endothelin, cathelicidin, and VIP), metabolites (prostaglandins D2, E2, leukotrienes, and PAF), and growth factors (SCF, GM-CSF, β-FGF, NGF, PDGF, TGF-β, and VEGF). A detailed description of these factors and the literature on their discovery can also be found in excellent review papers authored by de Silva et al., 2014 and Moon et al., 2014 [13,14]. It should also be added that the content of granules depends on the tissue from which MC was isolated. Mast cells contain a variety of mediators (as mentioned above), such as heparin, histamine, tryptase, chymase, VEGF, and TNF-α, which, when released during the initial stages of wound healing, affect bleeding, followed by coagulation and acute inflammation. Various additional vasoactive and chemotactic rapidly produced mediators (C3a, C5a, PAF) contribute to these processes, while mast-cell-derived pro-inflammatory and growth-promoting mediators (VEGF, PDGF, TGF-β, NGF, IL-4, IL-8) contribute to neoangiogenesis, fibrinogenesis, or re-epithelialization during the repair process [15].

The secretion of substances accumulated inside the mast cell granules occurs via exocytosis. Several pathways of transmembrane transfers were described [14,16], e.g.,

- anaphylactic exocytosis (degranulation without *de novo* synthesis), observed after treatment with C3a and C5a complement peptides (on complement receptors), snake venom, UV, acrolein, titanium nanoparticles;
- selective exocytosis (degranulation) of cellular granules can occur after the action of specific stimulators, including antigen or IgE on FcεR1 (histamine is released), neuropeptides on NKR (cytokines, chemokines, and 5HT are released), 48/80 on Mrgprx2 (cytokines and chemokines are released), cathelicidin on G-protein-coupled receptor (GPCR, releasing histamine), defensins on GPCR (releasing histamine), pleurocidin on GPCR (releasing cytokines and chemokines), A23187 (releasing cytokines and chemokines), morphine and codeine on opioid receptor (releasing cytokines, chemokines, and hexosaminidase), and NGF on Trk receptor (releasing histamine and PGE2);
- constitutive exocytosis is associated with granule release without degranulation after treatment with zymosan on TLR2 (releasing GM-CSF, Il-1β), poly (I-C) on TLR (releasing cytokines), LPS on TLR4 and CD14 (releasing cytokines and chemokines), SCF on MAPK (releasing cytokines), and lectin on TIM3 (releasing cytokines);
- without degranulation and without *de novo* synthesis (exocytosis of exosomes). Exosomes are vesicles measuring 30 nm–100 nm in diameter formed in secretory granules. These vesicles penetrate the cell membrane, pass outside the cell, and after transferring in body fluids, they are engulfed by other cells. Exosomes are important for intercellular communication. They contain many proteins, up to 1000 different mRNAs, and more than 100 microRNA molecules [17,18,19].

The biogenesis and release of cytoplasmic granule contents require the presence of many proteins, including Munc 13-4 [20], complexin (synaphin) [21], RAB GTPase [22], lysosomal trafficking regulator protein (LYST), synaptotagmin [23], granins, RAC1/2 [24], DOCK3 [25], proteoglycans, and sensitive factor attachment protein receptor (SNARE) [26].

**Figure 1:**
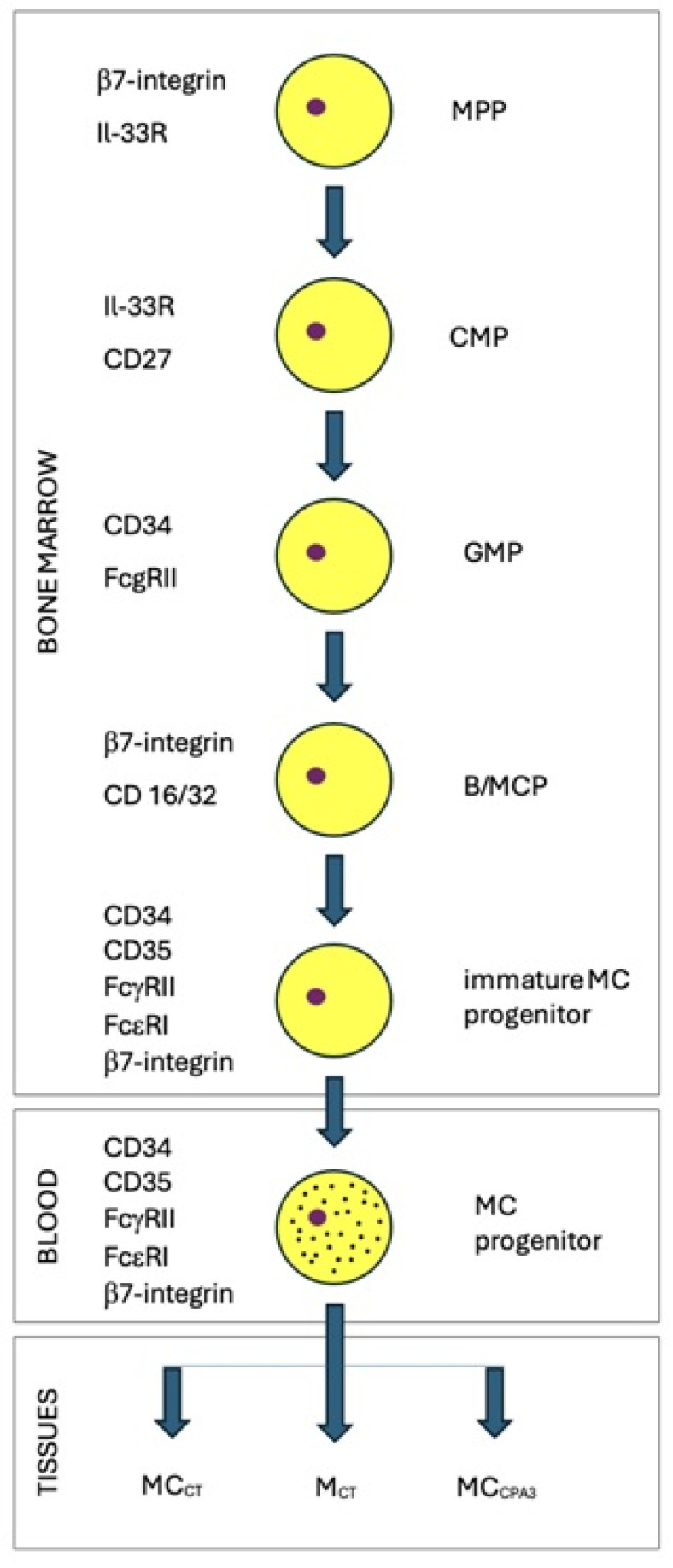
Stages of MC maturation. Erythroid–myeloid progenitors and hematopoietic adult stem cells were not included in the figure. MPP—multipotent progenitor; CMP—common myeloid progenitor; GMP—granulocyte-macrophage progenitor; B/MCP—basophil and MC progenitor; MC_CT_—containing tryptase, chymase, and CPA in the granules; M_CT_—containing only tryptase in the granules; and MC_CPA3_—containing CPA3 carboxypeptidase in the granules.

The origin of MCs—the effector cells of innate immunity—was initially attributed exclusively to myeloid hematopoiesis. It is now accepted that a certain amount of MC is derived from erythroid–myeloid progenitors (EMP cells) from the yolk sac [27]. The next stages of maturation in the bone marrow are (1) hematopoietic adult stem cells (HemASC); (2) multipotent progenitor containing β7-integrin, Il-33R, and active glycolysis (MPP); (3) common myeloid progenitor containing Il-7R, CD27, and active glycolysis (CMP); (4) granulocyte-macrophage progenitor containing CD34, FcγRII, and active glycolysis (GMP); (5) basophil and MC progenitor containing β7-integrin, CD16/32, and active glycolysis (B/MCP); (6) immature MC progenitor (possessing CD34, CD45, FcγRII, FcεRI, and β7-integrin, with active glycolysis); (7) MC progenitors (possessing CD34, CD45, FcγRII, FcεRI, β7-integrin, granules and glycolysis) (Figure 1). MC progenitors are present in peripheral blood and can mature in target tissues under the influence of cytokines, chemokines, and growth factors [28]. The stages of maturation are presented according to Mendoza et al., 2021 [29]. Images of mature mast cells using electron microscopy are shown in the paper by MacDonald et al. [30]. Five types of morphologically distinct granules were identified in the cytoplasm of MCs: (type I) electron-dense core surrounded by sparse particulates; (type II) less electron-dense and more electron-lucent core; (type III) uniform lumen/particulates; (type IV) a mixture of electron-dense vesicles; and (type V) particulates and scroll-like or multi-lamellar vesicles [30].

Two types of MCs have been described in rodents: mucosal mast cells (MMCs) and connective tissue mast cells (CTMCs). MMCs are found in the mucosal epithelia of the lungs and gastrointestinal tract. These cells contain the mMCP1 and mMCP2 proteases (chymases) bound to chondroitin sulfate. CTMCs are present in the submucosa of the intestines, peritoneum, and skin. These cells contain mMCP4 chymase, mMCP5 and mMCP6 tryptases, as well as mCPA carboxypeptidase bound to serglycine proteoglycans [31,32]. Three types of MCs have been described in humans: MC_CT_ containing tryptase, chymase, and CPA in the granules; M_CT_ containing tryptase alone [33,34]; and MC_CPA3_ containing CPA3 carboxypeptidase but not chymase. M_CTs_ are present in the mucous membranes of the intestines and lungs. MC_CCT_s are present in the skin, lymph nodes, and submucosa of the intestines and lungs. MC_CPA3s_ are observed in the epithelia of the lungs (in asthma) and esophagus (in eosinophilic inflammation) [35,36]. Mature tissue MCs are long-lived cells [37]. Few studies have been conducted on the differentiation and phenotypes of mature MCs in dogs.

Mast-cell-specific antigens are present on the surface of MCs—for example, CD16, CD 32, and other proteins (Table 1) [13,38,39,40,41,42,43,44].

### Mast Cell Ligands and Receptors

MCs respond to multiple ligands, which stimulate or inhibit the secretion of factors stored in their granules or synthesized *de novo*. Two types of degranulation are distinguished: total (anaphylactic) degranulation and fragmentary degranulation [14]. In addition, growth factors, prostaglandins, eicosanoids, chemokines, and cytokines are synthesized in MCs (especially after IgE stimulation) [45]. MCs can be alternatively stimulated by pathogen-associated molecular patterns (PAMPs) acting on Toll-like receptors, growth factors, complement peptides, cytokines, and other factors. They then selectively produce factors such as TNF-α [46], histamine [47], proteases [48], VEGF, PDGF-β, Il-6 [49], and Il-1 [50]. These factors act on blood vessels, on the intercellular matrix, on T cells, NK cells, macrophages, myeloid-derived suppressor cells (MDSCs), and dendritic cells [16].

MC ligands act by binding to cell membrane receptors. Stimulation of FcεRI by IgE is the main cause of allergic reaction [51]. IgE bound to the receptor recognizes various antigens. FcεRI stimulation involves phosphorylation by Fyn kinase of the receptor’s immunoreceptor tyrosine-based activation motif (ITAM) domain. This results in the binding and autophosphorylation of Syk kinase [52]. This leads to numerous protein phosphorylations, lipid metabolism, calcium ion mobilization, and activation of transcription factors [53].

Several types of mast cell receptors have been described—for example, SCF-binding receptor, PD1/CD279 receptor, Siglec-8, mas-related G-protein-coupled receptor member X2, Toll-like receptors, thymic stromal lymphopoietin receptor, and ATP receptors.

*KIT* is a proto-oncogene [54] and encodes a a receptor tyrosine kinase. Stem cell factor (SCF) activates the receptor and consequently many intracellular proteins are phosphorylated. KIT enables the proliferation of HemASC but also the survival and proliferation of differentiated mast cells [55], dendritic cells, and NK cells [56]. Eosinophils [57], and especially brain cells, also show high expression of KIT [58]. KIT expression depends on the presence of microphthalmia-associated transcription factor (MITF) and Gata2 transcription factor [59]. Upon SCF stimulation, KIT dimerizes and undergoes autophosphorylation in the juxtamembrane (JM) domain [60], kinase domain, and C-terminal domain. Further signaling proceeds through mitogen-activated protein kinase (MAPK), phosphoinositide 3-kinase (PI3K), phospholipase C gamma (PLC-γ), and JAK kinase [61]. KIT’s ligand is a stem cell factor (SCF), which has two isoforms: SCF220 and SCF248 [62]. SCF is synthesized by bone marrow stromal cells, eosinophils, fibroblasts, and smooth muscle cells [55].

The PD1/CD279 (programmed cell death protein) receptor is activated by ligands PDL1/CD274 and PDL2/CD273 [63].

The inhibitory receptor Siglec-8 is a sialoadhesin related to CD33. It was detected in 2000 and is expressed in MCs (but not MC precursors), eosinophils, and basophils. Its ligands are sialylated keratan sulfate, sialic acid, and glycans [64].

The mas-related G-protein-coupled receptor member X2 (MRGPRX, MrgprB2 in mice) is expressed in MCs (MC_CT_ in mice) present in the skin and subcutaneous tissue [65], as well as in eosinophils and basophils [66]. In MC_CT_ cells, degranulation is not dependent on FcεRI but on stimulation of MRGPRX by HDP (peptides for protection against bacterial infection), the cationic polymer C48/80, by opiates, or vancomycin [67]. Bacteria attacking the skin release quorum sensing molecules (QSMs) and competence sensing peptides (CSPs), which, through MRGPRX and MC_CT_ degranulation, cause bacterial growth inhibition [68].

MC-type cells have been detected in invertebrates, which lived over 500 million years ago [69]. Their granules contained heparin and histamine and degranulated upon stimulation. Invertebrate MC cells may represent the oldest “part” of the innate immune system.

Toll-like receptors (TLRs) bind pathogen-associated molecular patterns (PAMPs) and house dust mites (HDM), which cause airway inflammation [70]. The main ligands of these receptors are acylated lipopeptides (binding with TLR1); peptidoglycans, mucins, hemagglutinins, and mannans (binding with TLR2); double-stranded DNA (dsDNA) (binding with TLR3); lipopolysaccharide (LPS), mouse mammary tumor virus (MMTV) (binding with TLR4); flagellin (associated with TLR5); viral single-stranded RNA (ssRNA) (binding with TLR7 and TLR8); DNA CpG islands of bacteria and viruses (binding with TLR8) [71]. Upon TLR stimulation, mast cells produce Il-37, which inhibits inflammatory processes, dimerizing under tryptase and heparin. Il-37 is a ligand of Il-18R [72]. Mouse MCs of baboon bone marrow cells (BBMC), peritoneal-cell-derived mast cells (PCDMC), fetal-skin-derived mast cells (FSDMC) stimulated with LPS and peptidoglycan (PGN) synthesize a great number of interleukins, cytokines, chemokines, TNF-α, GM-CSF, INF-α, and INF-γ (partly depending on the stimulated TLR) [73,74,75].

Thymic stromal lymphopoietin receptor (TSLPR) binds thymic stromal lymphopoietin (TSLP), which is an alarmin released from respiratory epithelial cells. TSLP plays a role in chronic skin inflammation [76]. The other alarmins include Il-25 and Il-33 [77]. Il-33 binds the ST2/lI-lR4 receptor [78] and increases the survival of “cutaneous” MCs by stimulating the anti-apoptotic protein BCL-XL [79].

ATP receptors P2X4, P2X7, and P2Y1 degranulate mouse MCs upon ATP binding, and adenosine receptors A1, A2a, A2b, and A3 (bound to G proteins) are located on the surface of lung MCs [80].

Mast cell pathology is characterized by the occurrence of degranulation. The best known is type I hypersensitivity reaction with IgE antibodies, which bind harmless antigens and Fc receptors on MCs, causing degranulation of MCs. The release of mediators can lead to a variety of effects, including edema, vasodilation, and bronchoconstriction [81].

## 3. Mast Cell Activation Syndrome

Mast cell activation syndrome (MCAS) depends on excessive secretion of mast cell mediators after, among others, IgE stimulation [82]. The congenital causes of MCAS are systemic mastocytosis (SM) and hereditary alpha-tryptasemia (HalfaT). Hereditary alpha-tryptasemia is caused by the presence of additional copies—in the form of duplication or amplification—of the tryptase-alpha gene (*TPSAB1* gene) [83,84,85].

MCAS can manifest as symptoms of local disease, including redness, pruritus, urticaria, and conjunctivitis; mild systemic disease symptoms, including pruritus, erythema, mild hypotension, mild dyspnea, and nausea; severe systemic disease symptoms, including epidermal blisters, angioedema, fever, sweats, severe shortness of breath, vomiting, diarrhea, and collapse; and chronic systemic disease symptoms, including atopic tissue inflammation, with symptoms lasting for an extended period of time.

MCAS is not a malignancy; there is no accumulation of large numbers of mast cells or specific gene mutations, although congenital mutations of the *IL13* gene have been observed in asthmatic patients [86].

Mast cells can also be independently stimulated by IgE via the receptor FcεR1; IgG through the receptor FcγR1 [87]; stem cell factor (SCF) via the receptor KIT/CD117 [88]; pathogen-associated molecular patterns (PAMPs) via TLR receptors [89]; lectins, dsRNA via TLR4 receptor, which releases TNF-α and INF-β without MC degranulation [90]; complement peptides via CR3, CR4, CR5 receptors [91]; and numerous other stimulators.

### 3.1. Systemic Mastocytosis

Systemic mastocytosis (SM) is a disease characterized by the accumulation of mast cells in organs and tissues [92]. In morphologically and immunotypically abnormal mast cells [93], *KIT* gene mutations are very often present [94].

Depending on the location, the disease is divided into

- cutaneous mastocytosis (CM)—most common in children—manifesting as urticaria pigmentosa as a diffuse or limited form of mastocytosis. Urticaria pigmentosa has good prognosis and usually resolves itself spontaneously;
- indolent SM (ISM), aggressive SM (ASM), bone marrow SM, mast cell leukemia (MCL) [95], as well as mast cell sarcoma [94];
- localized mastocytoma [95].

The classification of benign human systemic mastocytosis (CM and ISM) was described by Hartmann et al., 2016 [96]. The classification of malignant ASM was proposed by Pardanani et al., 2010 [97]. Neoplastic MCs show specific expressions of tryptase [98], CD2, and CD25 [99] and less specific expressions of CD117, CD33, CD43, and CD68 antigens [98]. The expression of CD2 and CD25 is diagnostically important because it is not present in normal mast cells [100,101]. The CD30/Ki-1 antigen (encoded by the *TNFRSF8* gene) is also present on the surface of malignant cells [102]. The soluble CD30 (sCD30) form in a concentration up to 130 ng/mL is present in the blood of patients with ASM and MCL [103].

Many chromosomal aberrations have also been described in systemic mastocytosis cells, including chromosome X monosomy (45,X), chromosome 7 monosomy (45,XX,-7 or 45,XY,-7), chromosome Y disomy (47,XYY), chromosome 8 trisomy (47,XX,+8 or 47,XY,+8), 46,del(12)(p13) [104]. Complex karyotypes are rarely observed [104].

The KIT receptor is constitutively expressed in mast cells. In human systemic mastocytosis (hSM), the *KIT* gene undergoes a frequent (in up to 80% of cases) p.D816V mutation in exon 17 [105]. However, the mutations are not present in the very early stages of the disease [106]. Other *KIT*-activating mutations, such as p.V560G, p.D815K, p.D816Y, p.D816H, and p.D820G, have been described, occurring in hSM in a total of 5% of all cases [107]. The blocking of KIT activity by PKC412 (midostaurin) inhibits cell proliferation in SM for only 3 years [108].

Three types of tropomyosin receptor kinases (TRKA, TRKB, and TRKC) and nerve growth factor receptor (NGFR) bind neurotrophins. The nerve growth factor (NGF) binds TRKA, a brain-derived neurotrophic factor (BNDF). Nuclear factor 4 (NF4) binds TRKB. Nuclear factor 3 (NF3) mainly binds TRKC [109]. Neurotrophins increase mast cell survival, function, and chemotaxis [110,111]. The activation of mouse TRK by tumor necrosis factor (TNF) and TRKB by BDNF quite often causes SM or even MCL [112]. TRK mutations (especially TRKB) activate tumor transformation and metastasis formation in mastocytosis [111].

Abnormal activation of the mammalian target of rapamycin (mTOR) complexes may play a role in mastocytosis [113]. Rapamycin (an mTOR kinase inhibitor) has been found to reduce the growth and viability of SM cells with the *KIT* p.F816V mutation [114].

Other gene mutations described in SM include *IDH1* and *IDH2* (encoding isocitrate decarboxylase) [115]; *ERK1*/*ERK2* in mice [116]; *SRSF2* and *SF3B1* (intron excision factor) [117]; *RUNX1* (intron excision factor) [118]; *KRAS* and *NRAS* [119]; *STAT5*, *AKT* [120]; *TET2* [121]; *SETD2* [122]; *ASX1* [123]. In most cases, genetic mutations are present in somatic cells. In SM some genetic changes may occur in the germline. These include substitutions in the *KIT* gene (p.K509I [124], p.A533D [125], p.N822J [126]) and substitution in the *IL4R* gene (p.Q576R [125]).

In SM, the pathogenic variants of the *CEBPA* gene (rs4616402) encoding a transcription factor, *TEX41* gene (rs4662380) encoding TEX41 lncRNA, and *TBL1XR1* gene (rs13077541) encoding a transducin-like protein 1 and associated with the X receptor were described [127].

#### Targeted Therapies

Understanding the role of MCs in cancer development and progression is critical for developing new targeted therapies for human cancers [128]. The relationship between the presence of MCs in tumors, the progression of angiogenesis, and tumor development may increase the possible role of MCs in cancer biology. Therefore, blocking the release of mediators with KIT receptor tyrosine kinase inhibitors (TKIs) (for example, imatinib, mastinib) may affect MC function [128,129,130], while blocking the release of mediators with tryptase inhibitors (gabexate mesylate and nafamostat mesylate, both of which are inhibitors of trypsin-like serine proteases) [128,131] can be an important therapeutic treatment for reducing tumor growth [132].

Imatinib mesylate (STI571) is a multi-kinase inhibitor approved for clinical use in the treatment of chronic myeloid leukemia, acute lymphoblastic leukemia (Philadelphia-positive), gastrointestinal stromal tumors (CD117-positive), and myeloid/lymphoid neoplasms with *PDGFR* gene rearrangements [133]. The *KIT* wild-type receptor is taken into account as imatinib target. The data on in vitro and in vivo efficacy of imatinib in *KIT*-mutated SM have shown contrasting results [134,135,136,137,138]. Some rare types of *KIT* mutants, as well as their wild-type alleles (encoding extracellular and juxtamembrane domain), have been proven to be imatinib sensitive under in vitro conditions. In contrast, cells with the most common *KIT* gene mutation p.D816V (in the kinase domain) are not sensitive to the effects of imatinib [134,135,136,137,138]. The response to imatinib relies on the presence of imatinib-sensitive mutations involving *KIT* (e.g., juxtamembrane or transmembrane *KIT* mutations) or *PDGFR* (e.g., *FIP1L1/PDGFRA* rearrangement) rather than on the absence of p.D816V *KIT* gene mutation [134,135,136,137,138].

In addition to imatinib, both preclinical and clinical trials have analyzed the role of other TKIs. Specifically, masitinib has been shown to have in vitro activity against PDGFR, Lyn tyrosine kinase, Fyn tyrosine kinase, and wild-type KIT [139]. Clinical studies of masitinib in patients with mastocytosis have focused mainly on exploring its potential utility for treating MC mediator-associated symptoms [140,141]. Midostaurin (PCK412) is a multi-kinase inhibitor, which competitively binds to the ATP binding site in the catalytic domain of tyrosine kinases, resulting in their inhibition. In addition to its activity against FLT3, it inhibits both wild-type KIT and KIT with p.D816V mutation, as well as other protein kinases, such as kinase insert domain-containing receptor (KDR), fibroblast growth factor receptor (FGFR), vascular endothelial growth factor receptor 2 (VEGFR2), FIP1L1/PDGFR fusion protein, and members of the serine/threonine protein kinase C (PKC) family [142]. Another selective KIT inhibitor with high affinity for p.D816V mutant KIT is avapritinib (BLU-285) [143]. Ripretinib (DCC-2618) is a novel type II tyrosine switch control inhibitor for the treatment of *KIT*-mutated cancers, including gastrointestinal stromal tumors (GISTs). BLU-263 is an inhibitor of KIT p. D816V with minimal central nervous system penetration compared to avapritinib [144]. Another example of a highly selective TKI is bezuclastinib (CGT9486). It has potent activity against KIT p.D816V, and it does not affect the functions of other closely related kinases. Bezuclastinib has shown preliminary clinical activity and a tolerable safety profile in patients with advanced solid tumors, including GIST [145].

### 3.2. Canine Systemic Mastocytosis

Systemic mastocytosis in dogs is rarely described, and it is more often diagnosed as a less aggressive form of mastocytoma [146]. It usually presents as cutaneous papules distributed on the trunk, head, perineum, and extremities as cutaneous mastocytosis (CM) or pigmented urticaria with Darier’s sign [147]. The infiltrates of well-differentiated mast cells are present in the dermis. The cells show KIT/CD117 expression, but usually, there are no mutations in the *KIT* gene and other genes typical for human mast cells [148].

The cytoplasmic granules of mast cells contain histamine, cathepsin G, chymase, tryptase, carboxypeptidase, proteoglycans (heparin, chondroitin sulfate), TNF, interleukins (such as Il-1, Il-4, Il-5, Il-6, and Il-13), and chemokines (CCL3 and CCL4) [149]. Degranulation results in pruritus, redness, and swelling of the skin, less often in bleeding and also vomiting, diarrhea, and coughing [147].

## 4. Mastocytoma

Mastocytoma (MCT) is common in animals such as dogs, cats, and mice, and it accounts for 7–21% of skin cancers in these animals, but it is quite rare in humans. Mastocytoma is a neoplasm, which occurs most often in older dogs (8–10 years old) as a single skin nodule or subcutaneous tissue. The lesions are located mainly in the skin and subcutaneous tissue, and they are less common in the gastrointestinal tract, spleen, liver, bone marrow, and the nervous system. Tumors in areas other than the skin and subcutaneous tissue are usually metastatic lesions. However, the literature has reported a case of mast cell tumor diagnosis in the nasal cavity [150]. Mastocytomas present in the skin are usually present in the form of tumors, which may be devoid of hair. Swelling and redness of the skin may appear around the tumor. Importantly, mast cell tumors can also show rapid infiltrative growth [151].

Mastocytomas can be most broadly divided into minimally malignant and highly malignant. The clinical classification of MCTs was first presented by Bostock DE [152] and followed by Patnaik AK [153], as well as Kiupel AM [154].

Histopathological tumor examination is crucial for making a complete diagnosis, developing a treatment plan, and assessing prognosis. There are two commonly used grading scales for cutaneous mast cell tumors: one created by Patnaik and another by Kiupel. The grading according to Patnaik is a three-stage scale, where grade I defines well-differentiated tumors confined to the dermis; grade II denotes tumors of intermediate differentiation, extending into the subcutaneous tissue; grade III denotes tumors of low differentiation, infiltrating the subcutaneous tissue [155]. One definite disadvantage of this method is its subjectivity. Ten histopathologists evaluated the same slides from 60 mast cell tumors [156]. Agreement among pathologists was at the level of 62.1% [156]. Kiupel developed a two-stage classification, which defines skin tumors as benign (low-grade) or malignant (high-grade). High-grade lesions are characterized by karyomegaly in at least 10% of the examined cells or at least seven mitotic figures, three multi-nucleated cells, or three atypical nuclei in ten view fields [157]. Kiupel’s classification is more authoritative in assessing the malignancy and the potential for metastatic lesions [155]. According to the Kiupel scale, the average survival time for patients with benign tumors is more than 2 years, while for patients with malignant tumors, the average survival time is less than 16 weeks [154]. Many laboratories continue to report both classifications in their test results to aid the cancer therapy process. The classification of mast cell tumors according to both Patnaik and Kiupel is not applicable to histopathological evaluation of subcutaneous mastocytoma [158]. Another prognostic factor is the number of mitoses in ten fields of view. It is believed that with the number of mitoses below seven, the prognosis is good, while the number of mitoses above seven corresponds to an average survival time of 12 weeks [159].

MCT tumors contain a large number of well-differentiated, highly granular tumor cells, a small number of eosinophils and cancer-associated fibroblasts (CAFs)—especially abundant in Patnaik’s grade III—lymphocytes, and other cells. Neoplastic MC cells have enlarged cell nuclei with scattered chromatin [160]. Nuclei and sparse mitotic figures are visible in these neoplastic cells [161].

Interestingly, malignant MCTs (grade I in Patnaik’s scale) in young dogs can remain inactive for several years and even involute [161]. Patnaik’s grade I at diagnosis applied to 20% of MCTs, grade II—43.3%, and grade III—36.7%. According to the Kiupel scale, there were 43.33% benign and 56.67% malignant MCTs [162]. Surgical removal within safe limits often yields durable results. Grade II tumors have a survival time of up to 4 years, while grade III tumors have a survival time of 1–2 years [162].

### 4.1. Diagnosis and Prognosis

Disease diagnosis should begin with a physical examination of the patient and clinical history taking.

Fine-needle aspiration biopsy (FNAB) is an effective tool in the process of initial diagnosis of mast cell tumors. By analyzing the patient’s data, the tumor location, and the result of FNAB examination, the tumor histological type of the mast cell can be predicted. Cutaneous mast cell tumors of the tail, perineum, or the site of skin transition to mucous membranes show high metastatic potential and are histologically qualified as malignant tumors [163].

Cytoplasmic granules contain negatively charged proteoglycans [164], proteases, chymase and tryptase, and carboxypeptidase A3 (CPA3) [165]. Hyaluronate breakdown products affect MC cell activation and migration. The procedure of choice is to remove the nodule with a margin of healthy tissue. The prognosis largely depends on local lymph node status with the use of the Ki67 expression assay [166], bromo d-uridine incorporation assay [166], proliferating cell nuclear antigen (PCNA) expression assay [167], AgNoR assay [168], MCT cell ploidy assay, MCT vascularization density assay [169], p53 expression assay [170], *KIT* gene mutation and expression assay [171], examination of the cell nuclei morphology [172], examination of cellular infiltration depth [173], and examination of tumor localization [174].

Evaluation of abnormal expression of tyrosine kinase receptor protein (KIT) by immunohistochemistry is an important prognostic factor, since the tyrosine kinase receptor protein plays a key role in mast cell proliferation, survival, differentiation, and migration. Three patterns of KIT expression have been distinguished. The first pattern (the membranous pattern, peritumoral) involves non-tumorigenic cells, as well as well-differentiated mast cell tumors. It indicates a non-aggressive biological type of tumor [175]. The second pattern (focal cytoplasmic pattern) shows focal or striated cytoplasmic labeling. In contrast, diffuse cytoplasmic labeling is observed in the third pattern. Both the second and third patterns are associated with shorter survival times and a higher risk of local relapse [175]. Immunohistochemical detection of phosphorylated KIT in patients with MCTs may predict the prognosis and biological behavior [176].

At the diagnosis of mast cell tumors, an analysis of the nuclear protein Ki67, which is a marker of cell growth fraction, is also conducted. At the same time, AgNOR nuclear proteins, visualized by silver staining, are the markers of cell division rates. A high number of AgNOR nuclear proteins indicates an increased cell cycle rate. Therefore, Ki67 assessment, along with AgNOR, is an important prognostic factor in the evaluation of cutaneous mastocytoma. An AgNOR × Ki67 equation score greater than 54 correlates with an increased risk of metastasis or death [177]. Smith et al. described AgNOR × Ki67 values as determinants of low cell proliferation in grade II MCTs [178].

Staging assessment should always be performed to determine the stage of disease development, which has a direct impact on therapeutic decisions and prognosis [179]. To assess the cancer grade, it is helpful to examine lymph nodes for the presence of cancer cells, even if there are no palpable changes in these lymph nodes. However, selecting the appropriate lymph node can present many difficulties. Therefore, it is helpful to perform lymph node mapping. As a result of manipulations associated with surgical tumor removal, mast cell granulomas may degranulate and recruit non-cancerous mast cells into the surrounding lymph nodes. This is the reason why lymph node biopsy is recommended before surgical removal of the lesion [180]. In order to evaluate the lymph nodes, a fine-needle aspiration biopsy can be applied, or the entire node can be harvested for histopathological evaluation. This approach allows the introduction of an appropriate therapeutic protocol [20]. Patients with possible metastases should additionally undergo liver and spleen biopsy. Infiltration with cancerous mast cells is possible even if the liver and spleen show normal morphology on abdominal ultrasound. Significant difference in mean survival time has been found between dogs with metastatic lesions in the liver or spleen and those whose organs are free of tumor cells [181].

### 4.2. Mastocytoma Cell Proteins

Several proteins of mastocytoma cells, which play important roles in the processes of their proliferation, migration, and resistance to apoptosis, have been described. MCT cell surface contains KIT/CD117, IgE, CD11b, CD18, CD44, and CD45, and sometimes CD2, CD25, and CD34 [182]. Other mastocytoma cell proteins are described in Table 2.

### 4.3. Mutations in Mastocytoma Cells

KIT/CD117 receptor mutations were studied as the typical changes for human mastocytosis. In 80% of human cases, the *KIT* gene substitution p.D816H is present. Loss of the KIT protein function may be the cause of human piebaldism syndrome [214]. The human KIT receptor has four isoforms [215]. The activating mutations described in the tumors result in dimerization of KIT, its multiple phosphorylation, and the generation of proliferative signal transmitted via the MAPK–PI3K–Akt–PLCg–JAK–Src pathway [61]. This creates an opportunity to interrupt cell proliferation signals by using multiple inhibitors of these enzymes [216].

*KIT* gene mutations in canine mastocytoma occur in 20–30% of cases [217]. Internal tandem duplication (ITD) in exon 11 includes nucleotides 555–559 and 571–590. Other common changes include deletions of nucleotides 550–560 [218] and point mutations of exon 11 (p.K557insF, p.K557insP). In exon 8, ITDs of nucleotides 417–421 or point mutation p.Q430R were observed. In exon 9, two mutations in the form of substitutions were found: p.S470I and p.N508I [219]. An increase in the *KIT* gene copy number is often found in canine mast cell tumors [220].

Some *KIT* mutations occur in healthy dogs, which have never been affected by MCT. However, there are dog breeds, which are highly susceptible to MCT, such as boxers, golden retrievers, Labradors, and bull terriers. This may suggest the existence of germline mutations in these specific breeds (Table 3).

### 4.4. Differences in Benign and Malignant Forms of MCT (According to Kiupel)

To date, few comparisons have been made between MCT cells in the benign and malignant forms of the disease. Studies have mainly focused on mutation specificity and differences in gene expression in the two forms of the disease.

ITD in exon 11 of the *KIT* gene is observed more frequently in malignant cutaneous MCTs with short survival and poor prognosis [227,228], while ITD in exon 8 of the *KIT* gene predicts longer survival and a milder course of the disease [229]. ITDs are also observed in exons 9, 12, and 19 of the *KIT* gene [219].

Certain cytogenetic changes in MCT cells are observed in aggressive mast cells. These include deletions in chromosomes 5, 20, and 21 and insertions in chromosome 21 [220]. Copy number variants (CNV) in the form of *PTEN* and *FAS* gene deletions, as well as amplifications of *MAPK3*, *WNT5B*, *FGF*, *FOXM1*, and *RAD51* genes, are also prognostically unfavorable [230].

It seems most important to compare the total gene expression in cells of malignant MCT forms with that of benign MCT forms. A study carried out on canine Agilent DNA arrays showed overexpression of 450 genes and lower expression of 140 genes in MCTs with a low differentiation degree [231]. The mRNA level of the *GSN* gene was particularly reduced in MCTs with a low differentiation degree. Overexpression of *FOXM1*, *GSN*, *FEN1*, and *KPNA2* genes was prognostically unfavorable. The authors selected 13 genes, whose expression levels most differentiated between the malignant and benign forms of MCT (Table 4).

Subsequent comparisons of gene expressions in high-risk and low-risk MCT cells were performed utilizing the Illumina platform [242]. Differential expression was found in 71 genes, of which 68 genes had increased expression in the high-risk group, and only 3 genes had decreased expression in this group. The results supported the conclusions of studies carried out previously. In addition, an important role was found to be played by cancer-associated fibroblasts (CAFs), which produce tumor intercellular matrix proteins [243]. CAF cells in the matrix activate tumor growth and progression. Tumor-associated fibroblasts (TAFs) are similar to myofibroblasts [244], and anti-smooth muscle antibodies (ASMAs) are the markers of these cells [245].

### 4.5. Metastasis Formation in Mastocytoma

Metastasis is observed in about 25% of MCTs, and its formation is a multi-step process caused by changes in gene expression [246]. Genes whose expression is associated with metastasis include those encoding proteins involved in intercellular adhesion and cell–ECM binding. Downregulation of their expression facilitates metastasis [246]. The expression patterns of such genes (in humans) have been described by Daves et al. [247]. Genes with a significantly reduced expression in metastatic cancers are listed in Table 5.

Overexpression of certain genes promotes metastasis, including the *DNAJ3A3* gene from the heat shock protein (HSP) family, which promotes tumor invasion, and the small nucleolar RNA, C/D Box 93 (*SNORD93*) gene, which blocks the expression of metastasis genes [259].

The release of a cancer cell from a primary tissue is part of the metastasis formation process. Tumor cells are transferred via lymphatic vessels to regional lymph nodes and via blood vessels to distant tissues. Tumors release cells into the bloodstream, but only cells reaching “premetastatic niches (PMNs)” can survive and proliferate. These PMNs are formed by cancer cells, inflammatory cells, cancer-associated adipocytes (CAA), tumor-associated macrophages (TAM), and cancer-associated fibroblasts (CAFs) derived in part from adipose-derived stem cells [260]. There are three types of macrophages: M1 (inhibiting and killing cancer cells), M2 (increasing metastasis formation) [261], and TAM [262].

### 4.6. The Role of microRNAs

Epigenetic changes refer to mechanisms connecting the genome with environmental signals to provide adaptations to various conditions, factors, and intrinsic pathologic processes [263]. There are various different types of epigenetic regulation, including DNA methylation, post-transcriptional modifications by microRNAs (miRNAs), and histone modifications [264].

MicroRNAs are small RNAs involved in the regulation of mRNA transcription [265]. MicroRNAs can circulate freely in the plasma, or they can be delivered by extracellular vesicles (EVs) and the small extracellular vesicles (sEVs) [266,267]. Extracellular vesicles are released by all cell types, including normal cells and tumor cells. Moreover, they are present in body fluids, including plasma, urine, milk, sweat, tears, saliva, and cerebrospinal fluid [268]. EVs play a role in cell-to-cell communication. They are able to carry different types of RNA, proteins, lipids, and even DNA fragments [269]. The compounds carried by EVs can affect the function of recipient cells [269]. Cancer cells communicate not only with each other but also with the surrounding cells, including immune cells, fibroblasts, and endothelial cells. This communication is possible through EVs, which play an important role in tumor progression [270]. Tumor EVs can affect the cell phenotype, and they can also affect cells in the microenvironment, which support tumor cell growth, their survival and local invasion, as well as metastasis [271].

MicroRNAs with higher expression in the exosomes (membrane-bound extracellular vesicles of MCT cells) in comparison to mast cells included hsa-miR-451, hsa-miR-503, miR-Plus_27560, miRPlus_2843, miRPlus_27564, hsa-miR-583, miRPlus_1795, miRPlus_17890, hsa-miR-663, and hsa-miR-30b [272]. The expression level of circulating sEV-miR-21-5p changes in the plasma of dogs in different pathological stages (MCT with or without nodal metastasis) [273]. The level of sEV-miR-21-5p was significantly higher in plasma collected from nodal metastatic MCT-affected dogs compared to healthy and early metastatic MCT-affected patients [273].

Deregulation in microRNAs expression is typical for various types of cancers. MicroRNAs can act as tumor suppressors or oncogenes. Their significance as prognostic or predictive factors in human and veterinary medicine has been reported by He et al., Agarwal et al., and Jain et al. [274,275,276]. The activation of murine mast cells and upregulation of the miR-221-222 family influence cell cycle checkpoints [277]. For example, miR-221 regulates mast cell degranulation, cytokine production, and cell adherence [278]. Fenger et al. found that *miR-9* was significantly overexpressed in aggressive MCTs compared to benign MCTs [253].

The miRNAs profile from paraffin-fixed tissues of canine MCT has been characterized by Zamarian et al. [279]. The panel of three miRNAs, including *miR-21-5p*, *miR-379,* and *miR-885*, has a good efficiency in discriminating healthy and MCT-affected dogs, as well as MCT-affected dogs with and without nodal metastasis [279]. Moreover, in the saliva of dogs affected by MCT, *miR-21-5p* has been identified as a potential negative prognostic factor [280]. *MiR-21-5p* is one of the miRNAs described as upregulated in canine MCT [279]. It was one of the first miRNAs detected in humans as an oncomiR [281]. Its overexpression is associated with oncogenesis in different tumors [281].

Lee et al. found that the expression of *miR-539* and *miR-381* is repressed by a mutated KIT protein with p.D816V substitution. Normally, these miRNAs are involved in *MITF* gene expression suppression. As a result, melanocyte-inducing transcription factor is not present within the cell. Dysregulation of the *miR-539* and *miR-381* pathways may contribute to abnormal MC proliferation and to the development of aggressive MC diseases [282].

### 4.7. Treatment

Mast cell tumors are the most common skin cancer in dogs. The disease forces veterinarians to consider various treatment options, including surgical treatment, pharmacological treatment, and radiotherapy. The obtained results should be analyzed carefully, based on the patient’s condition. The most important issue is to assess the risk of local relapse and metastasis, and only on this basis select an adequate management protocol.

#### 4.7.1. Surgical Treatment

One of the treatment options for mast cell tumors is surgical removal of the lesion [283]. Surgery is usually a fully therapeutic procedure, as long as an adequate surgical margin is maintained. A study population of 55 dogs, after complete removal of grade II mast cell tumor, showed recurrence or metastasis in 5% of cases [284]. However, *de novo* tumor development was observed in 11% of the studied dogs [284]. Achieving clean margins during surgical resection can often be the most difficult aspect. Moreover, the histopathological margins’ evaluation also depends on the technique used to deliver the tissue sample to the laboratory. It is important to ensure that markings of the section are made accurately. This should be achieved by using special ink or surgical threads. Attention should also be paid to maintaining accuracy when filling out the laboratory referral, as it can facilitate an accurate diagnosis for the doctor examining the lesion. Sometimes, mast cell tumors are accompanied by swelling and redness, and there may be mast cells in the tissue surrounding the tumor, which have not undergone neoplastic transformation. The doctor examining the section must distinguish between healthy mast cells and tumor-transformed mast cells. Radial sections are routinely used to make a histopathological diagnosis and to assess the margin of healthy tissue in mast cell tumors. Although this method has been shown to be effective in making a diagnosis, it can lead to difficulties when evaluating the lesion margins. In contrast, tangential sections are a much more sensitive method when assessing whether the margin of healthy tissue has been preserved. Radial sections, based on palpation of the slice, provide good-quality specimens, which indicate general information about the surgical margins. Tangential sections allow a more accurate assessment of the margins. By combining the two methods, the sensitivity in detecting “dirty” margins is 20% [285].

Assessing the risk of local relapse is important for the subsequent therapeutic process. Therefore, diagnostics can be further expanded with molecular testing [178]. The local relapse risk of low-grade tumors—according to Kiupel’s scale, with a low index of Ki67 or AgNOR × Ki67—is less than 10%. At the same time, about 40% of “high-grade” tumors with a mutation in exon 11 of the *KIT* gene are at risk of relapse, despite maintaining clean margins [178]. The higher number of AgNORs in silver-stained nuclei regions is associated with higher proliferation of tumor-transformed cells and the degree of tumor transformation. The number of AgNORs may be a predictor of tumor recurrence [286]. The molecular changes in cancer cells undergoing metastasis are still poorly understood. In the *KIT* mutational status (in exons 8, 9, and 11), 100% concordance was observed between primary and metastatic MCTs in twenty-one prospectively enrolled canine patients [287]. In contrast, ITD mutations were present in the primary tumors and were not found in relative metastasis [288].

#### 4.7.2. Pharmacological Treatment

Pharmacological treatment is used primarily in patients in whom complete surgical resection is impossible or multiple metastases are found. In addition, therapeutic substances can be used in neoadjuvant therapy. The use of glucocorticoids in skin mastocytoma therapy makes it possible to nullify symptoms associated with degranulation of granulomas, such as swelling or redness of the skin. Glucocorticosteroids administered orally have a cytotoxic effect against tumor-transformed mast cells, and their use has few side effects [289]. Glucocorticosteroids inhibit the rate of tumor growth and slow tumor cell divisions. However, in the absence of appropriate receptors, resistance to glucocorticosteroids can develop [290].

Chemotherapy, despite its side effects, effectively controls the disease and extends the average survival time in dogs with mastocytoma [291]. Therapy with Lomustine (CCNU) has shown limited efficacy. In a group of twenty-three dogs with MCT at various stages, according to Kiupel’s classification, one dog showed complete response; seven dogs had a partial response; and in six dogs, the disease remained stable [292]. Vinblastine belongs to the group of cytostatics. It stops cell division in the metaphase. Moreover, it can lead to apoptosis of non-dividing cells, damage of tumor vascularization, and disruption of translation. Treatment regimens using vinblastine are used in patients in advanced disease stages, when the tumor is inoperable, and in dogs with cutaneous mast cell tumors with a high degree of malignancy [293].

Targeted therapy involves the administration of chemical compounds, which block tumor growth through their effects on specific molecules, such as enzymes and proteins. Targeted therapy is less harmful to normal cells and may have fewer side effects in comparison to conventional chemotherapy. Letard et al. showed that about 20–30% of mast cell tumor cells have a mutation in the KIT receptor, which is responsible for mast cell growth and differentiation [219]. In veterinary medicine, toceranib was the first drug approved for targeted treatment in animals, and it is still indicated for canine mast cell tumors [294,295,296]. It is a drug, which simultaneously targets multiple receptor tyrosine kinases (for example, vascular endothelial growth factor receptor (VEGFR), PDGFR, KIT). Another TKI approved for the treatment of canine mast cell tumors is masitinib mesylate, which, when administered orally, allows MCTs with a mutation in the KIT receptor to inhibit growth. In a study by Letard et al., dogs which received masitinib showed significantly longer survival times relative to the placebo group but only among patients with a mutated KIT receptor [219,291]. Masitinib has been shown to inhibit KIT and other tyrosine kinase receptors, such as PDGFRs and fibroblast growth factor receptor 3 (FGFR3) [139,294]. In their meta-analysis review paper, Coehlo and colleagues pointed out that, among dogs treated with TKIs, 257 dogs received the treatment under the label; 261 received the drug off-label; and 87 dogs received masitinib due to the presence of non-resectable mast cell tumors (grade II or III) with confirmed mutated KIT [133]. In the case of imatinib, this drug is not recommended in dogs [133]. Webster et al. found that treatment with vinblastine in combination with prednisone after surgery was beneficial for dogs with grade III MCT compared to those treated only with surgery. Moreover, dogs with *KIT* mutations, which were treated with this protocol, had a longer disease-free interval and survival duration [171]. In most cases of canine mastocytoma, molecular analyses are not carried out routinely; therefore, it is difficult to assess the effects of targeted treatment in these animals.

Electrochemotherapy is used in MCT treatment, which combines the intratumoral/intravenous application of specific drugs and treatment with electrical impulses [297]. Electrochemotherapy leads to temporary electropermeabilization of the cell membrane and entry into the cell of the chemotherapeutic agent to enhance its cytotoxic effect [297]. To perform this technique, two main drugs are used: bleomycin (intratumoral application) and cisplatin (intravenous application) [298]. To prevent the effects of mast cell degranulation, it is important to apply electrical pulses initially at the periphery of the tumor, then move toward the center [179].

#### 4.7.3. Radiotherapy

Mast cell tumors, which have been removed with an adequate margin of healthy tissue, usually require no further treatment [284]. Unfortunately, the location of the tumor in the extremities, head, or tail area often prevents the preservation of so-called “clean” margins, and adjuvant therapy must therefore be implemented. Grade II or III mast cell tumors are treated with radiotherapy, which significantly reduces the risk of local relapse [299]. Mastocytomas respond very well to radiotherapy. In a radical protocol, fractionated irradiation is usually used. The average survival time for patients undergoing radiation therapy was between 2 and 5 years [300]. Radiotherapy treatment of mast cell tumors has additional applications as neoadjuvant treatment to facilitate complete tumor resection [300].

When using treatment with ionizing radiation, it is important to consider the possible side effects, which are divided into early and late reactions. Early side effects include swelling, redness, ulceration, and burning of the skin. Late reactions include leukotrichia, skin discoloration, and fibrosis within the irradiated area [301]. In extreme cases, it can lead to osteoradionecrosis, damage of lymphatic and blood vessels, lymphedema, as well as the formation of another tumor [302].

## 5. Conclusions

This review summarized the advancement in research on the genetic changes in mast cells, including point mutations (mainly), gene expression, chromosomal aberrations, and epigenetic changes in the form of microRNA expression. The prognosis depends on the histologic type of the tumor and the level of progression in the body. Increasingly advanced diagnostic techniques, combined with modern therapeutic approaches, allow long survival times. Genomic knowledge can be applied to the practice; for example, *KIT* gene mutations are very often present in mastocytoma cells, which affects disease prognosis. KIT/CD117 receptor mutations are typical changes for human mastocytosis. In contrast, in systemic mastocytosis, high KIT/CD117 expression is observed, but usually, there are no *KIT* gene mutations. This suggests that epigenetic mechanisms may play a significant role in mastocytosis development and metastasis. Moreover, the expressions of some microRNA molecules (classified as epigenetic mechanisms) have been shown to distinguish healthy and MCT-affected dogs, as well as dogs with and without nodal metastasis. Our paper reviews studies on mast cells, mastocytoma, and mastocytosis carried out over several decades and summarizes the research results conducted in this area. Further studies on the pathobiology of mast cells can lead to clinical improvements, such as better diagnosis and treatment of individuals affected by MCT.

## Figures and Tables

**Table 1:** MC main cell membrane markers.

Marker	Type	Protein Function
CD16 (FcγRIII, FCGR3A)	Migration	Receptor
CD32 (FcγRII, FCGR2)	Migration	Receptor
CD34	Migration, Tissue specific	Adhesion molecule
CD63	Activation	Receptor
ENPP3 (CD203c)	Activation	Receptor
FCER1 (IgE receptor, FCεRI)	Maturation	Receptor
ITGA4 (integrin α4, CD49d)	Migration	Receptor
ITGB7 (integrin β7)	Maturation, Tissue specific	Receptor
KIT (CD117, c-Kit)	General	Receptor
VCAM1 (CD106)	Migration, Tissue specific	Receptor

**Table 2:** Proteins of mastocytoma cells.

Protein	Function
CD30/Ki-1 antigen	A marker of Hodgkin’s disease and anaplastic lymphoma kinase (ALK) cells [183]. It has been shown that CD30 is expressed in human mastocytosis cells. CD30 expression in canine mastocytosis is inhibited by Il-4, which inhibits MCT cell proliferation [184].
Ki67 protein (encoded by *MKI67* gene)	The Ki67 protein is a general marker of cancer cells. Its synthesis is limited to G2 and M cell cycle phases [185]. The Ki67 protein has two isoforms (320 and 359 kDa), both of which bind and stabilize mitotic chromosomes [186]. Ki67 is an important factor for cancer development [187].
WWOX protein (oxidoreductase with a WW domain)	WWOX is a tumor suppressor protein (46 kDa) in dogs [188] and mice [189]. The WWOX protein is involved in DNA repair after ionizing radiation [190]. In mastocytoma cells, the level of WWOX is always strongly reduced [188].
Histamine H1 receptor (HR1) antagonists	HR1 antagonists (loratidine, terfenadine) inhibit the proliferation and reduce the viability of mastocytoma cells. Desloraphidine, rupatadine, and cyproheptadine are particularly effective (in higher concentrations) [191].
Proliferating cell nuclear antigen (PCNA)	PCNA is involved in DNA replication and DNA repair. PCNA has a PCNA-interacting protein box (PIP box) motif and forms a trimer, which slides along the DNA chain [192]. In MCT, an increased expression of PCNA is a poor prognostic factor [193].
Integrins	MCT cells bind collagen, fibronectin, and intercellular matrix laminin via extracellular matrix (ECM) β1 and α1-6 integrins [194]. In particular, VLA5 integrins are the activators of MCT [195]. Changes in ECM are generated by cancer-associated fibroblast (CAF) cells, and the structure of ECM in mastocytoma is similar to the structure of ECM in the stroma of a healing wound [196].
Hsp32 (heme oxygenase 1)	Hsp32 is synthesized by human cancerous mastocytes and dog MTCs [197]. Hsp32 protein is a cell survival factor. Its inhibitors include pegylated zinc protoporphyrin (PEG-ZnPP) and midostaurin, which inhibit tumor growth and induce apoptosis in human SM cells [198].
Hsp90	The protein is a chaperone, and its levels are elevated in tumors [199]. Hsp90 is also required for cancer cell survival [200]. Geldanamycin and its derivatives are the inhibitors of Hsp90 [201]. At low concentrations, Ganetespib (STA9090) induces the apoptosis of mastocytoma cells [202].
p53	The protein is expressed in mastocytoma cells, with the highest expression seen in Patnaik’s grade I and the lowest in grade II.
MCL1	The anti-apoptotic protein MCL1 of the BCL2 family is present in MCT cells (independent of *KIT* mutations) [203]. Downregulation of MCL1 protein expression in tumors increases their susceptibility to chemotherapy. In hematopoietic U937 cells, downregulation of MCL1 via antisense strategy causes apoptosis [204].
Programmed cell death ligand 1 (PD-L1)	The PD-L1 protein ligand of the programmed cell death 1 (PD-1) immunoinhibitory receptor is expressed in 66% of cases of mastocytoma and other canine cancers [205]. This is a poor prognostic factor. The PD-1 receptor causes T-cell infiltration of tumors, while PD-L1 inhibits T-cell function. Anti-PD-L1 antibodies reactivate T cells and increase IFN-γ production (also in human pancreatic cancer) [206]. The blocking of PD-L1 by antibodies can cause tumor regression [207].
Down syndrome cell adhesion molecule (DSCAM)	Cell surface protein DSCAM is important for nervous system development. Mutations in humans are also associated with Down syndrome, Hirschsprung’s disease, and idiopathic scoliosis [208]. The DSCAM gene of Labrador dogs is located on chromosome 31; its mutations increase the risk of MCT up to 1.66 times. Alterations in the DSCAM gene have been described in 40 types of different cancers [209].
Proteins involved in hyaluronic acid metabolism	Hyaluronic acid metabolism genes (in golden retrievers) contain nucleotide polymorphisms on chromosome 14 (for *HYAL4*, *HYALP1*, and *SPAM1* genes) and on chromosome 20 (for *IPK1*, *HYAL1-3*, and *GNAI2* genes) [210]. Defects in hyaluronate metabolism in the skin are responsible for the risk of MCT in Shar-Pei dogs [211]. Hyaluronate breakdown products cause mast cell activation and migration [212].
Multi-drug resistance protein 1 (MDR1)	The *MDR1* gene encodes a glycoprotein, which is part of the pump responsible for removing foreign substances from the cell. It also removes exogenously administered drugs previously taken up by the cell. Inhibition of *MDR1* gene expression may be useful in chemotherapy [213].

**Table 3:** Germline genetic variants in mastocytoma.

Gene/DNA Segment	Genetic Variant/Type of Mutation
*KIT*	Germline mutations in the *KIT* gene are observed infrequently, rather involving cancers other than MCT. Only *KIT* gene ITD mutations in exon 11 can be germline in MCT [221].
*TP53*	*TP53* mutations are present in 14.6% of MCTs [222].
*GNB1*	In dogs, mutations in *GNB1* have been found in cutaneous and subcutaneous MCTs, with a trend toward positive prognosis [223].
*DSCAM*	Genetic variant rs850678541 inhibiting DSCAM protein synthesis [224].
Single-nucleotide polymorphisms (SNPs)	SNPs in the *HYAL1-4*, *SPAM1*, and *GNAI2* genes play a role in mast cell tumor development [210].
*MCL1*	Overexpression of the myeloid target leukemia (MCL1) gene is observed in many cancers, including in mastocytoma. It encodes a labile BCL2 family protein located in the mitochondria [225].
Mitochondrial D-loop sequence	D-loop mutations in mitochondrial DNA are present in 47% of dogs with MCT and are usually homoplasmic. Six haplotypes of mitochondrial DNA sequences have been described in MCT cells [226].

**Table 4:** Expression of genes differing between malignant and benign forms of MCT.

Gene	Product
*CCNB*	Cyclin B is important for the transition from the G2 phase to mitosis. *CCNB* is an oncogene, which is important in the process of metastasis [232].
*FOXM1*	Encodes a transcription factor with a forkhead domain. *FOXM1* has high expression in proliferating tumor cells [233].
*CDC20*	Encodes a karyokinetic spindle protein. *CDC20* is an oncogene, which can initiate apoptosis [234].
*CDCA8*	Encodes a regulator of mitosis in the centromeric CPC complex.
*NUF2*	Encodes a protein of the NDC80 complex in the kinetochore. Silencing its expression results in apoptosis [235].
*NUSAP1*	Encodes a karyokinetic spindle protein, which determines the survival of cancer cells [236].
*PRC1*	Encodes a protein regulator of cytokinesis, which is present in the G2 and M phases. Overexpressed in neurons [237].
*CENPP*	Encodes a centromere protein, plays a role in kinetochore function and during mitosis [238].
*UBE2S*	Encodes ubiquitin-conjugating enzyme and plays a role in mitosis [239].
*GSN*	Encodes an anti-oncogene, which plays a role in apoptosis. The only gene with lower expression in MCT and in many other cancers [240].
*FEN1*	Encodes an endonuclease, which plays a role in DNA synthesis and is an anti-oncogene [241].

**Table 5:** Downregulated genes in metastatic cancers.

Genes	Product
Keratin genes	Genes encoding keratin, mainly *KRT1*, *KRT5*, and *KRT15* genes [248].
*SDPR*	Serum deprivation response protein. *SDPR* is a metastasis suppressor gene [249].
*NME1*	Co-transcription of this gene and the neighboring downstream gene (*NME2*) generates naturally occurring transcripts (NME1-NME2), which encode a fusion protein comprising sequences sharing identity with each individual gene product [250].
*SHARP1*	bHLH transcription factor [251].
*LIFR*	Leukemia inhibitory factor receptor [252].
*PERP*	TP53 apoptosis effector. It presents low expression in mouse MCTs overexpressing miR-9 [253].
*SBSN* and *SFN*	SBSN (suprabasin) and SFN (stratipin).
*PSORS1C2*	Epithelial cell protein.
*CLEC3B*	C-type lectin domain family 3 member B. It is an ECM biomarker protein for metastasis.
*EGR1*	Early growth response 1 gene. Its product blocks heparanase, which increases metastasis.
*CD9*	Encodes a four-transmembrane protein, which blocks cell motility [254].
*BRAF* and *ADFN*	The *BRAF* gene encodes serine/threonine kinase. The *ADFN* gene encodes multi-domain protein involved in signaling and the organization of cell junctions during embryogenesis. BRAF and ADFN deficiency increases cell motility.
*EVL*, *ARHGEF10*, *NF2*	Their products activate stress fiber formation [255].
*SCRIB*	Encodes scribble planar cell polarity protein.
*PKP1* and *DSP*	Desmosome proteins plakophilin 1 (PKP1) and desmoplakin (DSP). Their absence promotes metastasis [256].
*SDC1*	Encodes a protein, which connects the cytoskeleton to the ECM [257].
*PMP22*	Encodes peripheral myelin protein 22. Its overexpression decreases cell motility [258].

## Data Availability

Not applicable.

## Abbreviations

5HT	5-hydroxytryptamine
ALK	anaplastic lymphoma kinase
ASM	aggressive systemic mastocytosis
ASMAs	anti-smooth muscle antibodies
B/MCP	basophil and MC progenitor
BBMC	baboon bone marrow cells
BNDF	brain-derived neurotrophic factor
CAA	cancer-associated adipocytes
CAF	cancer-associated fibroblast
CAFs	cancer-associated fibroblasts
CCL	C-C motif ligand
CM	cutaneous mastocytosis
CMP	common myeloid progenitor
CNV	copy number variants
CPA3	carboxypeptidase A3
CSF-1R	colony-stimulating factor receptor
CSPs	competence sensing peptides
CTMCs	connective tissue mast cells
DOCK3	dedicator of cytokinesis 3
DSCAM	Down syndrome cell adhesion molecule
DSP	desmoplakin
dsRNA	double-stranded ribonucleic acid
ECM	extracellular matrix
EMP	erythroid–myeloid progenitors
EV	extracellular vesicles
FGF	fibroblast growth factor
FNAB	fine-needle aspiration biopsy
FSDMC	fetal-skin-derived mast cells
GM-CSF	granulocyte-macrophage colony stimulating factor
GMP	granulocyte-macrophage progenitor
GPCR	G-protein-coupled receptor
HalfaT	hereditary alpha-tryptasemia
HDM	house dust mites
HDP	heme detoxification protein
HemASC	hematopoietic adult stem cells
HR1	histamine H1 receptor
hSM	human systemic mastocytosis
HSP	heat shock protein
Il	interleukin
ISM	indolent systemic mastocytosis
ITAM	immunoreceptor tyrosine-based activation motif
ITD	internal tandem duplication
JM domain	juxtamembrane domain
LPS	lipopolysaccharide
LYST	lysosomal trafficking regulator protein
MAPK	mitogen-activated protein kinase
MCAS	mast cell activation syndrome
MCL	mast cell leukemia
MCP4	monocyte chemoattractant protein 4
MCs	mast cells
MCT	mastocytoma
MDR1	multi-drug resistance protein 1
MDSCs	myeloid-derived suppressor cells
miRNA	micro-ribonucleic acid
MITF	microphthalmia-associated transcription factor
MMCs	mucosal mast cells
MMTV	mouse mammary tumor virus
MPP	multipotent progenitor
NF3	nuclear factor 3
NF4	nuclear factor 4
NGF	nerve growth factor
NGFR	nerve growth factor receptor
NK	natural killer
NKR	natural killer receptor
PAF	platelet-activating factor
PAMPs	pathogen-associated molecular patterns
PCDMC	peritoneal-*cell*-derived mast cells
PCNA	proliferating cell nuclear antigen
PD1/CD279	programmed cell death protein
PDGF	platelet-derived growth factor
PDGFR	platelet-derived growth factor receptor
PD-L1	programmed cell death ligand 1
PEG-ZnPP	pegylated zinc protoporphyrin
PGE2	prostaglandin E2
PGN	peptidoglycan
PI3K	phosphoinositide 3-kinase
PKP1	plakophilin 1
PLC-γ	phospholipase C gamma
PMNs	premetastatic niches
QSMs	quorum sensing molecules
SCF	stem cell factor
SDPR	serum deprivation response
sEV	small extracellular vesicle
SM	systemic mastocytosis
SNARE	sensitive factor attachment protein receptor
ssRNA	single-stranded ribonucleic acid
TAFs	tumor-associated fibroblasts
TAM	tumor-associated macrophages
TGF	transforming growth factor
TIM3	T-cell immunoglobulin and mucin domain 3
TLRs	Toll-like receptors
TNF	tumor necrosis factor
Trk	tropomyosin receptor kinase
TSLP	thymic stromal lymphopoietin
TSLPR	thymic stromal lymphopoietin receptor
VEGF	vascular endothelial growth factor
VEGFR	vascular endothelial growth factor receptor
VIP	vasoactive intestinal peptide
